# Parkinson’s Disease Diagnostic Observations (PADDO): study rationale and design of a prospective cohort study for early differentiation of parkinsonism

**DOI:** 10.1186/s12883-018-1072-x

**Published:** 2018-05-16

**Authors:** Anouke van Rumund, Marjolein B. Aerts, Rianne A. J. Esselink, Frederick J. A. Meijer, Marcel M. Verbeek, Bastiaan R. Bloem

**Affiliations:** 1 Radboud university medical center, Department of Neurology, Donders Institute for Brain, Cognition and Behaviour, P.O.Box 9101, 6500 HB Nijmegen (935), The Netherlands; 2 Radboud university medical center, Department of Radiology and Nuclear medicine, Donders Institute for Brain, Cognition and Behaviour, P.O.Box 9101, 6500 HB Nijmegen (766), The Netherlands; 3 Radboud university medical center, Department of Laboratory Medicine Nijmegen, Donders Institute for Brain, Cognition and Behaviour, P.O.Box 9101, 6500 HB Nijmegen (830), The Netherlands

**Keywords:** Parkinson’s disease, Atypical parkinsonism, Cerebrospinal fluid, MRI, Differential diagnosis

## Abstract

**Background:**

Differentiation of Parkinson’s disease (PD) from the various types of atypical parkinsonism (AP) such as multiple system atrophy (MSA), progressive supranuclear palsy (PSP), dementia with Lewy bodies (DLB), corticobasal syndrome (CBS) and vascular parkinsonism (VP), can be challenging, especially early in the disease course when symptoms overlap. A major unmet need in the diagnostic workup of these disorders is a diagnostic tool that differentiates the various disorders, preferably in the earliest disease stages when the clinical presentation is similar. Many diagnostic tests have been evaluated, but their added value was studied mostly in retrospective case-control studies that included patients with a straightforward clinical diagnosis. Here, we describe the design of a prospective cohort study in patients with parkinsonism in an early disease stage who have an uncertain clinical diagnosis. Our aim is to evaluate the diagnostic accuracy of (1) detailed clinical examination by a movement disorder specialist, (2) magnetic resonance imaging (MRI) techniques and (3) cerebrospinal fluid (CSF) biomarkers.

**Methods/design:**

Patients with parkinsonism with an uncertain clinical diagnosis and a disease course less than three years will be recruited. Patients will undergo extensive neurological examination, brain MRI including conventional and advanced sequences, and a lumbar puncture. The diagnosis (including level of certainty) will be defined by a movement disorders expert, neuroradiologist and neurochemist based on clinical data, MRI results and CSF results, respectively. The clinical diagnosis after three years’ follow-up will serve as the “gold standard” reference diagnosis, based on consensus criteria and as established by two movement disorder specialists (blinded to the test results). Diagnostic accuracy of individual instruments and added value of brain MRI and CSF analysis *after* evaluation by a movement disorder expert will be calculated, expressed as the change in percentage of individuals that are correctly diagnosed with PD or AP.

**Discussion:**

This study will yield new insights into the diagnostic value of clinical evaluation by a movement disorder specialist, brain MRI and CSF analysis in discriminating PD from AP in early disease stages. The outcome has the potential to help clinicians in choosing the optimal diagnostic strategy for patients with an uncertain clinical diagnosis.

**Trial registration:**

NCT01249768, registered November 26 2010.

**Electronic supplementary material:**

The online version of this article (10.1186/s12883-018-1072-x) contains supplementary material, which is available to authorized users.

## Background

Differentiation between Parkinson’s Disease (PD) and the various forms of atypical parkinsonism (AP) can be difficult, especially early in the disease course. This is due to overlap in clinical symptoms, certainly when the clinical picture has not yet developed fully. Forms of AP such as multiple system atrophy (MSA), progressive supranuclear palsy (PSP) or corticobasal syndrome (CBS) follow a more malignant disease course than PD and, unlike PD, generally do not respond well to dopaminergic therapy.

While difficult, a correct diagnosis is important for adequate disease management and patient counseling, as well as for research purposes. If, in the future, any disease-modifying therapy becomes available, then reliable diagnostic tools will be essential to distinguish PD from AP at the earliest possible stage.

Because there is not yet a reliable objective test for PD and AP, expert opinion remains the gold standard for the clinical diagnosis. In the hands of a movement disorders specialist, the diagnostic accuracy is higher (90%) compared to judgment by a general neurologist (76%) [1, 2]. Although the clinical diagnosis after several years of follow-up correlates highly with the neuropathological diagnosis upon post-mortem examination, the diagnostic accuracy of the initial diagnosis varies greatly, and can be as low as 30% [1, 3–5]. When a clinician has little doubt about the diagnosis, ancillary investigations have hardly any diagnostic value. But when the clinical picture is puzzling, which is often the case early in the disease course, reliable biomarkers are needed for accurate and early differentiation between PD and AP.

Brain MRI is nowadays part of the diagnostic workup of patients with suspected parkinsonism, mainly to exclude secondary causes of parkinsonism (e.g. tumor, hydrocephalus, multiple sclerosis, vascular parkinsonism). Conventional MRI can demonstrate abnormalities characteristic for various AP syndromes, such as putaminal or olivopontocerebellar atrophy in MSA, midbrain atrophy in PSP and asymmetric regional cortical atrophy in CBS.

Due to limited sensitivity of these MRI abnormalities (e.g. 68% for cerebellar atrophy and 59% for putaminal atrophy), their contribution to the clinically based diagnosis is limited [6]. These abnormalities often occur late in the disease course, which make them of less diagnostic value in early disease stages. However, advances in neuroimaging techniques are promising to provide new biomarkers for improved differentiation between the different parkinsonian syndromes.

Magnetic resonance volumetry (MRV), diffusion tensor imaging (DTI), susceptibility weighted imaging (SWI) and resting state functional magnetic resonance imaging (rs-fMRI) are examples of new MR techniques which have become available [7–10]. However, these new techniques are often difficult to interpret for the individual patient, since MRI patterns are investigated on a group level. Moreover, validated diagnostic criteria are generally lacking. For example, DTI studies found alterations in fractional anisotropy and mean diffusivity in the substantia nigra in PD patients and in the cerebellum, pons, cerebellar peduncles and putamen in MSA patients. Furthermore, increased iron accumulation in the substantia nigra on SWI sequences is suggestive for PD as is increased putaminal hypointensity for MSA [7, 11, 12]. Rs-fMRI has been applied to assess functional abnormalities in neural networks of PD patients. Several studies identified PD-specific network patterns compared to healthy controls, but if these patterns differ from AP cases is yet unknown [13].

Furthermore, research on cerebrospinal fluid (CSF) biomarkers has strongly developed over the past decade. Analysis of brain specific proteins in CSF can be of value in distinguishing PD from AP. In PD patients, concentrations of brain specific proteins and neurotransmitter metabolites are generally within normal ranges, whereas in AP aberrant levels of these proteins and metabolites can be found. Currently, neurofilament light chain (NFL), total and phosphorylated tau (t-tau and p-tau), α-synuclein (α-syn) and amyloid-β42 (Aβ42) seem to be the most promising biomarkers for the differential diagnosis of parkinsonian syndromes. NFL levels are increased in AP compared with PD and healthy controls, tau proteins are elevated in CBS, while Aβ42 and tau are associated with cognitive decline (dementia) in PD and DLB [14–21].

Given the complexity of parkinsonian syndromes it is likely that neither a single clinical test nor a single biomarker can differentiate between the different parkinsonian syndromes. A combination of parameters could however improve diagnostic accuracy [14, 15, 18, 19, 21]. On the other hand, MRI is time-consuming and expensive, CSF analysis requires a lumbar puncture and many centers lack a neurologist with experience in movement disorders. More knowledge about the diagnostic value of each test could help clinicians in selecting the best available test.

With this prospective cohort study, we aim to gain insight in which single or combined ancillary investigation (including the opinion of a movement disorder specialist) will have the highest diagnostic yield for discrimination of PD and AP in early disease stage when diagnostic uncertainty is high.

## Methods/design

### Study design

A prospective observational cohort study to assess the diagnostic value of clinical evaluation by a movement disorder specialist, brain MRI and CSF analysis in discriminating different forms of parkinsonism early in the disease course. The Medical Ethics Committee Arnhem-Nijmegen approved the study. Written informed consent will be obtained from all patients after detailed explanation of the procedures.

### Objectives

The main objective of our study is to determine the diagnostic accuracy of detailed clinical examination by a movement disorder specialist, conventional and advanced brain MRI techniques and CSF analysis to differentiate PD from AP in clinically uncertain cases. Secondary objective is to identify potential variables for differentiation between PD and AP.

Third objective is to identify potential discriminative variables between the various forms of AP.

### Study population

Patients will be recruited from our outpatient movement disorder clinic and outpatient neurology clinics of referring general hospitals. Inclusion criteria are clinical signs and symptoms of parkinsonism (hypokinetic-rigid syndrome) with uncertain clinical diagnosis and disease duration less than three years. Exclusion criteria are age below 18 years and unstable co-morbidity. Inclusion and exclusion criteria are provided in Table 1.

**Table 1:** Inclusion and exclusion criteria

Inclusion criteria	Exclusion criteria
Hypokinetic-rigid syndrome of neurodegenerative origin Disease duration < 3 years Age > 18 years	Instable comorbidity Incompetent patient A medical history of brain surgery Other neurodegenerative disorder Diagnosis of *clinically established* or *probable* PD Diagnosis of *probable* MSA, PSP, CBS or VP
	*Exclusion criteria for MRI:* Claustrophobia Metal devices Unable to lie still for 30 min Pregnancy
	*Exclusion criteria for lumbar puncture:* Coagulation disorders Oral anticoagulants

*PD* Parkinson’s disease, *MSA* multiple system atrophy, *PSP* progressive supranuclear palsy, *CBS* corticobasal syndrome, *VP* vascular parkinsonism

### Clinical examination

#### Evaluation by a movement disorder specialist

All patients will be interviewed and examined by a movement disorder specialist at the outpatient neurology department of the Radboud university medical center in Nijmegen (The Netherlands).

#### Structured interview

Standardized questionnaires on demographics, medical history, cardiovascular risk factors, activities in daily living, medication use, disease onset, clinical signs, most affected body site, balance and fear of falling will be administered at baseline and every year during follow-up for 3 years. In addition, the Non-Motor Symptom Scale (NMSS) [22], Hospital Anxiety and Depression Scale (HADS), Questionnaire for Impulsive-Compulsive Disorders in Parkinson’s Disease (QUIP) and Epworth Sleepiness Scale (ESS) will be scored [23–25]. A translated version of the structured questionnaires are provided as Additional file 1 with this article.

#### Cognitive assessment

A measurement of global cognitive function will be assessed by the Mini Mental State Examination (MMSE) [26], Frontal Assessment Battery (FAB) [27] and Cambridge Neuropsychological Automated Test Battery [28] at baseline and after three years follow-up.

#### Neurological examination

Standard neurological examination will be performed including testing of cranial nerves, motor and sensory function, tendon reflexes and presence or absence of primary reflexes. Horizontal and vertical blood pressure will be measured to screen for orthostatic hypotension.

In addition, the following clinimetric scales will be assessed: Unified Parkinson’s Disease rating scale (UPDRS) III and IV, Hoehn and Yahr disease severity score [29] and Scale for the assessment and rating of ataxia (SARA) [30]. The neurological examination will be performed and videotaped at baseline and after three years follow-up.

### Ancillary investigations

#### MRI

All patients will have a brain MRI at first presentation performed on 3 Tesla MRI scanner (Siemens Magnetom Trio, Erlangen, Germany) by a standardized scanning protocol. Conventional MRI sequences will include 3D T1 MPrage, T2 turbo-spin echo, T2 fluid attenuated inversed recovery (FLAIR), and proton density sequences. Advanced MRI sequences will include diffusion weighted imaging (DWI) and diffusion tensor imaging (DTI), SWI and rs-fMRI. All MRI scans will be evaluated in a systematic fashion by two neuroradiologists blinded for clinical symptoms and outcome. The presence of the following abnormalities on conventional MRI sequences will be scored using a diagnostic workstation (Agfa-Impax version 6.5.3, Gevaert, Mortsel, Belgium): atrophy and T2 hypo-intensity of the putamen, putaminal rim sign, frontal lobe and parietal lobe atrophy, lateral, third and fourth ventricle dilatation, midbrain and pontine atrophy, hummingbird sign, atrophy of the cerebellum and cerebellar vermis, atrophy of the medulla oblongata, T2 hyper-intense signal changes of the middle cerebral peduncle and hot cross bun sign, dilated perivascular spaces, lacunar infarctions and white matter changes. Advanced imaging sequences will be analyzed by dedicated software packages, including ‘Functional MR Imaging of the Brain Software Library‘(FSL, University of Oxford, United Kingdom) and ‘Statistical Parametric Mapping’ (SPM, Trust Centre of Neuroimaging, London, United Kingdom).

#### CSF

The following CSF variables will be analyzed according to previously described methods [15, 31–33]: Aβ40, Aβ42, t-tau, p-tau (phosphorylated at Thr181), α-syn, NFL, 3-methoxy-4-hydroxyphenylethyleneglycol (MHPG), 5-hydroxyindolacetic acid (5-HIAA), homovanillic acid (HVA), blood pigments and the total cell count. The cell count and blood pigments will be analyzed within 2 h after CSF collection; α-syn will be analyzed according to previously described methods [32]; all other parameters will be analyzed within 4 weeks after CSF collection. CSF samples are collected in polypropylene tubes, centrifuged, aliquoted, and stored at − 80 °C until analysis. Because the concentrations of HVA and 5-HIAA vary in the different fractions of CSF, we aim for separate collection of the 9th–11th ml fraction for analysis of neurotransmitter metabolites, according to previously described methods [34]. Laboratory technicians blinded for clinical symptoms and outcome will perform all CSF analyses. When patients are taking dopaminergic medication at the time of lumbar puncture, the results of neurotransmitter metabolites will be excluded from the measurements.

### Follow-up

During the three years after baseline examinations patients will be followed by their own neurologist. Every year patients are requested to complete a structured questionnaire. After three years, all patients will be re-examined by the study investigator (AR).

### Assessment of diagnoses

Before inclusion, the referring neurologist will be asked to establish the probable diagnosis based on routine clinical examination. Specifically, a level of diagnostic (un) certainty will be indicated on a scale of 0–100. After inclusion, a movement disorder expert will also be asked for the diagnosis and level of certainty based on the clinical evaluation; a neuroradiologist makes the diagnosis based on imaging findings and a neurochemist based on CSF analysis.

**Fig. 1:**
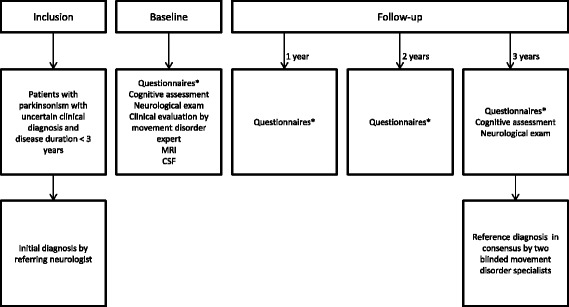
flowchart of the study design. *A translated version of the structured questionnaires are provided as Additional file 1 with this article

The final clinical diagnosis will be established after three years of follow-up in consensus by two independent neurologists specialized in movement disorders, and this will serve as the “silver standard” reference diagnosis. This reference diagnosis will be based on the clinical data at baseline, the disease course after 3 years follow-up and with adherence to the following international clinical criteria: Movement Disorder Society clinical diagnostic criteria for PD [35], Gilman criteria for MSA [36], the movement disorder society criteria for PSP [37]**,** Armstrong criteria for CBS [38] and Zijlmans criteria for vascular parkinsonism [39]**.** These neurologists will be blinded for the results of the ancillary investigations, except for the conventional MRI because this is part of routine procedures in clinical practice. See Fig. 1 for a flowchart of the study design.

### Outcome measures

Our primary outcome measure of interest will be the diagnosis (e.g. PD or AP) after three years of follow-up. The secondary outcome measure is the specific diagnosis (PD, MSA, PSP, etc.) after three years of follow-up.

### Statistical analysis

For our first objective, the estimated initial diagnosis by the movement disorder specialist, neuroradiologist and neurochemist respectively will be compared to the reference diagnosis (after three years follow-up). Sensitivity, specificity and Kappa (k) are calculated. Next, the Net Reclassification Index (NRI) is calculated, which gives the proportion of participants correctly (re-)classified as having PD or AP. NRI values will be calculated for MRI and CSF *following* the classification by a movement disorder specialist [40].

For our second objective, descriptive data will be generated for all variables. Continuous variables will be presented as means ± standard deviations or medians. PD patients will be compared with AP patients using a Student’s t or Mann-Whitney U test, whichever appropriate.

Dichotomous variables will be compared using Chi-square test or Fisher exact test, whichever appropriate. A *p*-value of < 0.05 will be considered significant (two-sided).

For each diagnostic strategy (e.g. clinical evaluation, MRI and CSF respectively) blockwise logistic regression with stepwise forward model selection will be used to identify parameters with optimal diagnostic power. For each model ROC-curves and C-statistics are calculated.

Data management and analysis will be performed using IBM SPSS Statistics 22 (IBM Corp, Armonk, NY, USA).

### Sample size calculation

This study is exploratory in nature. We estimate that a minimum of 30 AP and 30 PD patients will be needed to evaluate the diagnostic value of the three diagnostic strategies (clinical evaluation by a movement disorder specialist, MRI and CSF biomarkers). In our previous cohort study of patients with parkinsonism 60% of patients had a diagnosis of AP [34]. 30% of patients did not complete the follow-up period of 3 years, mainly because the participant had deceased or was too severely affected, the majority of which was diagnosed with AP (33 out of 46 drop-outs). Therefore, we estimate that approximately 100 participants will be needed to assess the diagnostic accuracy of the different diagnostic tests.

### Loss to follow-up

In case patients opt to withdraw from the study or are no longer able to participate, the investigator will try to obtain a final interview and neurological examination if possible. When loss to follow-up is greater than 20%, analysis will be done to check for associated biased effects.

## Discussion

A major unmet need in the diagnostic workup of parkinsonian disorders is a diagnostic tool to differentiate the various forms of parkinsonism when the clinically based uncertainty is high. This study is especially designed to provide directions for the neurologist in daily practice when the clinical picture of parkinsonism is puzzling.

Recently, we completed a similar prospective study of 156 consecutive patients with parkinsonism, but with an initially uncertain diagnosis [34]. All patients underwent extensive clinical testing and the following ancillary investigations: routine brain MRI; 123I-iodobenzamide single photon-emission computed tomography (IBZM-SPECT); CSF analysis; and anal sphincter electromyography (EMG).

We have shown that clinical tests such as tandem gait analysis and the axial UPDRS sub-score had a high predictive value to discriminate between PD and AP [34]. NFL and tau in CSF differentiated MSA from PD and tau and p-tau protein were elevated in CBS. Nonetheless, none of the ancillary investigations, (routine brain MRI, IBZM-SPECT, CSF analysis and anal sphincter EMG) was superior to the clinical judgment in differentiating parkinsonian syndromes [34].

However, diagnostic uncertainty in this previous cohort study was defined as being present when the neurologist who referred the patient was uncertain about the diagnosis (even when the patient fulfilled the probable criteria for one of the parkinsonian syndromes). Furthermore, patients from all disease stages were included. Therefore, the proportion of patients in an early disease stage and with an actual uncertain diagnosis was relatively small.

For that reason, the a priori chance that ancillary investigations would be of additional value was limited. Moreover, no advanced MRI techniques were used. Thus, we designed this present study to assess the diagnostic value of ancillary investigations in a population of patients with parkinsonism and an unclear clinical picture at the beginning of their disease course. We included the judgment of a movement disorder expert in the baseline examinations to compare its added diagnostic value to the various ancillary investigations. As ancillary tests, we chose brain MRI (both conventional and advanced MRI techniques) and CSF collection because of their potential to reveal new biomarkers for differentiating PD from AP based on developments over the past decade.

The gold standard for diagnosis of PD and the AP remains neuropathological confirmation of the clinical diagnosis upon post-mortem examination. However, prior research has demonstrated that neuropathological data show a high concordance between the clinical diagnosis after three years follow-up established by a movement disorder specialist and neuropathological examination post-mortem [1]. Therefore, we will use the clinical diagnosis after three years follow-up as reference diagnosis, or ‘silver standard’, with which we compare the results of ancillary investigations in individual patients. In order to minimize the potential misdiagnosis that might occur using the clinical diagnosis instead of the neuropathological diagnosis, we require the clinical diagnosis to be established in consensus by two neurologists highly experienced in movement disorders and according to the international criteria.

This study will help the general neurologist select the best available diagnostic “tool” in differentiating parkinsonian syndromes (e.g. clinical evaluation by a movement disorder specialist, brain MRI or CSF biomarkers).

## Additional file

Additional file 1: Structured questionnaire English translation. English translation of the annual questionnaire. Description of data: English translation of the annual questionnaire of the Parkinson’s Disease Diagnostic Observations Study (PADDO). (PDF 758 kb)

## Abbreviations

AP: atypical parkinsonism
Aβ40: amyloid-β40
Aβ42: amyloid-β42
CBS: corticobasal syndrome
CSF: cerebrospinal fluid
DTI: diffusion tensor imaging
MRI: magnetic resonance imaging
MRV: magnetic resonance volumetry
MSA: multiple system atrophy
NFL: neurofilament light chain
PD: Parkinson’s disease
PSP: progressive supranuclear palsy
p-tau: phosphorylated tau protein
rs-fMRI: resting state functional magnetic resonance imaging
SWI: susceptibility weighted imaging
t-tau: total tau protein
UPDRS: Unified Parkinson’s Disease rating scale
α-syn: α-synuclein
