# Supplementary material for: Parkinson’s Disease Diagnostic Observations (PADDO): study rationale and design of a prospective cohort study for early differentiation of parkinsonism
Source: BMC Neurol. 2018 May 16;18:69. doi: 10.1186/s12883-018-1072-x (PMC5954463; doi:10.1186/s12883-018-1072-x)
Supplement: Supplementary file 1 — Structured questionnaire English translation. English translation of the annual questionnaire. Description of data: English translation of the annual questionnaire of the Parkinson’s Disease Diagnostic Observations Study (PADDO). (PDF 758 kb) [file 12883_2018_1072_MOESM1_ESM.pdf]

PADDO
Parkinson's Disease Diagnostic Observations

This is the annual questionnaire of the Parkinson's Disease Diagnostic Observations Study (in short: PADDO). Could you please fill out the questions below?

Demographics

Date of birth (day/month/year): ..../.........../................

Gender: M / F

Disease course

What was the course of your motor symptoms in the past year?

1. Gradual unilateral decline (only left or right body side)
2. Gradual decline on both body sides
3. Rapid unilateral decline (only left or right body side)
4. Rapid decline on both body sides
5. Stepwise decline
6. My symptoms are stable, no decline

Do you experience choking with food, liquid or both?

0. Never
1. Approximately once a year
2. Approximately once a month
3. Approximately once a week
4. Approximately once a day

Have you ever developed an aspiration pneumonia due to choking?

0. No
1. Yes

Have you been admitted to a hospital in the past year?

0. No
1. Yes, because of ....
   ....
   ....

PADDO Parkinson's Disease Diagnostic Observations

Please check if the form is complete

Activities of daily living and mobility

Where do you live?

1. Independent, in my own house
2. In a care home/home for the elderly
3. In a nursing home

Are you able to do normal activities of daily living without help (e.g. bathing/showering, grooming, dressing, etc.) ?

1. Completely independent
2. With help from spouse, family or other informal caregiver
3. With help from professional caregiver

Are you able to perform your job or hobbies?

1. Yes, without difficulties/impairments
2. Partially, with adjustments
3. Not able to perform my job or hobbies

Do you use a walking stick?

0. No, never
1. Yes, since (month/year) ……………………./……………………….
2. I used to use one, since (month/year) ……………………./………………………., but not anymore

Do you use a walker?

0. No, never
1. Yes, since (month/year) ……………………./……………………….
2. I used to use one, since (month/year) ……………………./………………………., but not anymore

Do you use a wheelchair?

0. No, never
1. Yes, since (month/year) ……………………./……………………….
2. I used to use one, since (month/year) ……………………./………………………., but not anymore

PADDO Parkinson's Disease Diagnostic Observations

Balance

Do you experience a tendency to fall?

0. No
1. Yes

How often do you fall (both falls and near-falls)

0. Never
1. Approximately once a year
2. Approximately once a month
3. Approximately once a week
4. Approximately once a day

When has this tendency to fall started?

0. Not applicable
1. Within 1 year after onset of Parkinson symptoms
2. Within 3 years after onset of Parkinson symptoms
3. More than 3 years after onset of Parkinson symptoms

Were you ever hurt or injured after falling?

0. Never
1. Yes, once
2. Yes, more than once

What were the consequences of your fall?

0. No consequences or no falling
1. Superficial bruises, scrapes or contusion of body parts
2. Fracture
3. Loss of consciousness or concussion (head injury)
4. Other type of injury:…………………………………………………………

What is usually the direction of falling?

0. No falling
1. Forward
2. Backward
3. Sideward
4. All directions/no particular direction

PADDO Parkinson's Disease Diagnostic Observations

Do you experience fear of falling?

0. No
1. Slight (does not influence my daily activities)
2. Moderate(moderate impact on my daily activities)
3. Severe (severe impact on my daily activities)

Do you ride a bicycle?

0. Never or stopped long ago (before Parkinson symptoms occurred)
1. Not anymore, since (month/year) ……………………../…………………
2. Yes, still

PADDO
Parkinson's Disease Diagnostic Observations

Please check if the form is complete

List of medication

Could you please fill your current medication in the list below according to the example?

Name medication: paracetamol
Dose (in mg per tablet/capsule/puff/etc.): 500 mg
Directions or daily dosage: 4 times a day 2 tablets

1. Name medication:…………………………………………………………………………………………………
   Dose: ………
   Directions or daily dosage:…………………………………………………………………………………

2. Name medication:…………………………………………………………………………………………………
   Dose: ………
   Directions or daily dosage:…………………………………………………………………………………

3. Name medication:…………………………………………………………………………………………………
   Dose: ………
   Directions or daily dosage:…………………………………………………………………………………

4. Name medication:…………………………………………………………………………………………………
   Dose: ………
   Directions or daily dosage:…………………………………………………………………………………

5. Name medication:…………………………………………………………………………………………………
   Dose: ………
   Directions or daily dosage:…………………………………………………………………………………

6. Name medication:…………………………………………………………………………………………………
   Dose: ………
   Directions or daily dosage:…………………………………………………………………………………

7. Name medication:…………………………………………………………………………………………………
   Dose: ………
   Directions or daily dosage:…………………………………………………………………………………

8. Name medication:…………………………………………………………………………………………………
   Dose: ………
   Directions or daily dosage:…………………………………………………………………………………

PADDO
Parkinson's Disease Diagnostic Observations

Please check if the form is complete

Anxiety and depression

[For an English version of this section of the questionnaire is referred to the Hospital Anxiety and Depression Scale (HADS): Zigmond AS, Snaith RP. The hospital anxiety and depression scale. Acta Psychiatr Scand. 1983;67:361-70.]

PADDO
Parkinson's Disease Diagnostic Observations

Freezing

Freezing is a temporary, involuntary inability to move. It may feel like your feet are stuck in place, or it may be difficult to get up from a chair. Usually, freezing only lasts a few seconds. It can happen at any time but tends to happen more often when you are initiating or starting to move (i.e. standing to walking), walking through doorways, turning or when surrounded by crowds. Please, encircle the answer that best applies to you.

1. Have you experienced freezing episodes in the past month?
    0. No
    1. Yes

If your answer to question 1 is "No" you can skip the other questions about freezing (questions 2-10) and proceed to the next questionnaire.

2. How often do you experience freezing episodes?
    0. Less than once a week
    1. Sometimes, approximately once a week
    2. Often, approximately once a day
    3. Very often, two or more times a day

3. How often do you experience freezing on turning?
    0. Never
    1. Rarely, approximately once a month
    2. Sometimes, approximately once a week
    3. Often, approximately once a day
    4. Very often, two or more times a day

If your answer to question 3 is "Never" you can proceed to question 5.

4. How long does your *longest* freezing episode on turning last?
    1. (less than) 1 second
    2. 2-5 seconds
    3. 5-30 seconds
    4. I'm not able to walk within 30 seconds

5. How often do you experience freezing on starting to move?
    0. Never
    1. Rarely, approximately once a month
    2. Sometimes, approximately once a week
    3. Often, approximately once a day
    4. Very often, two or more times a day

Please check if the form is complete

PADDO Parkinson's Disease Diagnostic Observations

If your answer to question 5 is "Never" you can proceed to question 7.

6. How long does your *longest* freezing episode on starting last?
   1. (less than) 1 second
   2. 2-5 seconds
   3. 5-30 seconds
   4. I'm not able to walk within 30 seconds

7. What is the impact of freezing on your mobility?
   0. No impact at all
   1. Slight
   2. Moderate
   3. Severe

8. Do you experience fear of falling due to freezing?
   0. Not at all
   1. Slight
   2. Moderate
   3. Severe

9. Are you able to walk without assistance?
   0. Yes, I can walk anassisted
   1. I need supervision, but no assistance
   2. I sometimes require some assistance
   3. I often require assistance
   4. I am unable to walk

10. What is the impact of freezing on your daily activities?
    0. No impact at all
    1. Slight, I avoid or am unable to do some activities
    2. Moderate, I avoid or am unable to do several daily activities
    3. Severe, I avoid or am unable to do most of my daily activities

PADDO Parkinson's Disease Diagnostic Observations

Please check if the form is complete

Impulse control

[For an English version of this section of the questionnaire is referred to the Questionnaire for impulsive-compulsive disorders (QUIP ANYTIME): Weintraub D, Mamikonyan E, Papay K, Shea JA, Xie SX, Siderowf A. Questionnaire for Impulsive-Compulsive Disorders in Parkinson's Disease-Rating Scale. Mov Disord. 2012;27:242-7.]

PADDO
Parkinson's Disease Diagnostic Observations

Please check if the form is complete

Sleep

[For an English version of this section of the questionnaire is referred to the Epworth Sleepiness Scale (ESS): Johns MW. A new method for measuring daytime sleepiness: the Epworth sleepiness scale. Sleep. 1991;14:540-5.]

PADDO
Parkinson's Disease Diagnostic Observations

Non motor symptoms

[For an English version of this section of the questionnaire is referred to the non-motor symptoms scale for Parkinson's disease (NMSS): Chaudhuri KR, Martinez-Martin P, Brown RG, Sethi K, Stocchi F, Odin P, et al. The metric properties of a novel non-motor symptoms scale for Parkinson's disease: Results from an international pilot study. Mov Disord. 2007;22:1901-11.]

PADDO
Parkinson's Disease Diagnostic Observations
